# NMR resonance assignments of the PR-10 allergens Act c 8 and Act d 8 from golden and green kiwifruit

**DOI:** 10.1007/s12104-021-10031-w

**Published:** 2021-06-09

**Authors:** Ricarda Zeindl, Martin Tollinger

**Affiliations:** Institute of Organic Chemistry, Center for Molecular Biosciences Innsbruck (CMBI), University of Innsbruck, Innrain 80/82, 6020 Innsbruck, Austria

**Keywords:** NMR resonance assignment, TALOS + prediction, PR-10 protein, Cross-reactivity, Allergen

## Abstract

Kiwifruits have become one of the most common food sources triggering allergic reactions. In patients suffering from birch pollen related food allergy, reactions result from initial sensitization to the birch (*Betula verrucosa*) pollen allergen Bet v 1, followed by immunological cross-reactivity to structurally homologous proteins in kiwifruit. Clinical symptoms range from scratching and itching of the oral cavity to more severe immunological reactions such as rhino conjunctivitis. In this work we assigned backbone and side chain ^1^H, ^13^C and ^15^N chemical shifts of the 17 kDa PR-10 allergens Act c 8.0101 and Act d 8.0101 from golden (*Actinidia chinesis*) and green (*Actinidia deliciosa*) kiwifruit by solution NMR spectroscopy. The chemical shift data confirm the characteristic Bet v 1 fold for both proteins, consisting of a seven-stranded antiparallel β-sheet interrupted by two short α-helices, along with a long C-terminal α-helix. Our data provide the basis for determining the three-dimensional solution structures of these proteins and characterizing their immunological cross-reactivity on a structural basis.

## Biological context

The most common allergy in Western and Central Europe is pollinosis, elicited by sensitization to birch pollen. About 70–90% of birch pollen allergic patients show IgE serum reactivity against the major birch pollen allergen Bet v 1 (Ipsen and Lowenstein 1983; Moverare 2002). Patients suffering from birch pollinosis frequently experience allergic reactions after consumption of certain fruits and nuts, due to immunological cross-reactivity of Bet v 1 specific antibodies (IgE) to proteins that are present in these food sources. Symptoms typically occur immediately after consumption and are mostly confined to the oral allergy syndrome, which includes itching and scratching of the throat and tongue (Mari et al. 2005). Around 50–60% of all individuals, who are sensitized towards birch pollen, exhibit allergic reactions after consumption of kiwifruit (Geroldinger-Simic, 2011; Le, 2013). Kiwifruit are of high nutritive and health value, with an exceptionally high vitamin C content (Hunter et al. 2011). Kiwifruit allergic patients avoid eating the fruit, thereby abstaining themselves from a valuable food source.

Immunological cross-reactivity to birch pollen is associated with the class 10 pathogenesis related (PR) proteins, Act c 8 and Act d 8, from golden (*Actinidia chinensis*) and green (*Actinidia deliciosa*) kiwifruit (Oberhuber, 2008). The expression of these proteins, which are believed to play a major role in plant defense, can be induced by pathogenic or environmental stress. PR-10 proteins consist of about 160 amino acid residues, with a molecular weight of 17–18 kDa. The canonical fold of PR-10 proteins consist of a seven-stranded antiparallel β-sheet (β1–β7), which is interrupted by two short α-helices (α1 and α2) between strands β1 and β2 and covered by a long C-terminal α-helix (Fernandes et al. 2013). Similar to other PR-10 proteins, Act c 8 and Act d 8 are easily degraded by proteolysis and are heat labile (Breiteneder and Mills 2005). In kiwifruit, the allergens Act c 8 and Act d 8 are located in the peripheral pulp. ELISA and immunoblot experiments showed that recombinantly produced Act c 8 and Act d 8 are recognized and bound by IgE in sera of birch pollen allergic patients in vitro, suggesting clinically relevant levels of immunological cross-reactivity with Bet v 1 (Oberhuber et al. 2008).

While the presence of multiple isoforms of Act c 8 and Act d 8, which may possess differential IgE binding potentials, is likely in kiwifruit, only a single isoform of each allergen has been identified so far. Among each other Act d 8.0101 (CAM31909) and Act c 8.0101 (CAM31908.1) share a sequence identity of 70%, while the sequence identity with the birch pollen allergen Bet v 1 is only 53% and 54%, respectively (Gajhede, 1996). The sequence identity of these two kiwi allergens with the most prominent cross-reactive food allergen, Mal d 1 from apple, is 57% and 58% (Ahammer et al. 2017, 2016). A peculiar feature of the two PR-10 allergens from kiwifruit is their unusually high number of cysteine residues.

In this work we present the solution NMR backbone and side-chain assignments of the two recombinantly expressed allergens Act d 8 and Act c 8.

## Methods and experiments

### Sample preparation

The codon-optimized inserts of Act c 8.0101 (GenBank nucleotide code AM489567.1 and protein code CAM31908.1) and Act d 8.0101 (GenBank nucleotide code AM489568 and protein code CAM31909) were each cloned in the expression vector pET28b (+) using restriction enzymes NcoI and XhoI. Transformation was conducted in the *E. coli* strain BL21(DE3) Star (Invitrogen). A starter culture (20 mL) of Luria Bertani (LB) medium with 25 µg/mL kanamycin was inoculated with one bacterial colony and incubated for 8 h at 37 °C and 200 rpm. 20 µL of the starter culture were transferred into 100 mL of M9 minimal medium and incubated overnight at 37 °C and 200 rpm. To reach a starting cell density of 0.1 in the final expression culture, the necessary volume of the overnight culture was calculated by V_o/n_ = (0.1 $\times$V_expression_)/A_600, o/n_. The appropriate volume was centrifuged at 2000$\times$*g* and the pellet was resuspended in 1 L of M9 minimal medium enriched with either ^15^NH_4_Cl (1 g/L) or ^13^C_6_-d-glucose (3 g/L) and ^15^NH_4_Cl (both Cambridge Isotope Laboratories) and supplemented with 25 µg/mL kanamycin. The culture was incubated at 37 °C and 200 rpm until the cell density reached 0.5–0.6 (at 600 nm), at this point, protein expression was induced by addition of isopropyl-β-d-1-thiogalactopyranoside (IPTG, 1 mM) and performed for 3 h at 37 °C. Cells were harvested at 4 °C and 4600$\times$*g* for 40 min, resuspended in a buffer containing 0.5 M urea, 25 mM imidazole and 0.1% Triton X-100 and stored until usage at -80 °C. For lysis, cells were thawed, treated for 40 min on ice with lysozyme (10 µg/mL) and DNAse (1 µg/mL), passed through a French Press and centrifuged at 16,000$\times$*g* and 4 °C for 45 min. The filtered (0.45 µm) lysate was loaded onto an anion exchange column (Resource Q 6 mL, GE Healthcare) and Act d 8.0101 or Act c 8.0101 protein was eluted with a sodium chloride gradient over 30 mL from 0 to 50% in 25 mM TrisHCl buffer (pH 7.5) at a flow rate of 2 mL/min. Fractions containing the desired protein were concentrated to about 1.5 mL by centrifugation (Amicon Ultra 3 kDa MWCO, Merck Millipore) and loaded onto a size exclusion column (HiLoad 16/600 Superdex 75 pg, GE Healtcare) for the final purification step and eluted isocratically at 1 mL/min with a 20 mM sodium phosphate buffer (pH 6.9). Both allergens eluted with a retention time that is in accordance with monomeric PR-10 proteins (Ahammer et al. 2016). SDS-PAGE gel electrophoresis was used to monitor all purification steps. NMR samples were prepared containing 0.5 mM ^15^N labeled and ^15^N/^13^C labeled protein, supplemented with 10% D_2_O (v/v). No reducing agents were added to NMR samples or during purification.

### NMR spectroscopy

All NMR spectra were recorded at 25 °C on 500 MHz Agilent DirectDrive 2 and 700 MHz Bruker Neo Avance spectrometers equipped with room temperature triple-resonance probes. A two-dimensional ^1^H-^15^N-HSQC and three-dimensional HNCACB, CBCA(CO)NH, HN(CA)CO and HNCO experiments were used to obtain the backbone resonance assignments. Additionally, a two-dimensional ^1^H-^13^C-HSQC and three-dimensional (H)CC(CO)NH-TOCSY, H(CCO)NH-TOCSY, ^1^H-^15^N-TOCSY-HSQC, ^1^H-^15^N-NOESY-HSQC and ^1^H-^13^C-NOESY-HSQC experiments were used for side-chain assignments. Aromatic side-chains of phenylalanines and tyrosines were assigned with aromatic ^1^H-^13^C-HSQC experiments. Data was processed with NMRPipe (Delaglio et al. 1995) and the CcpNMR software package was used for resonance assignment (Vranken, 2005).

### Assignments and data deposition

**Fig. 1:**
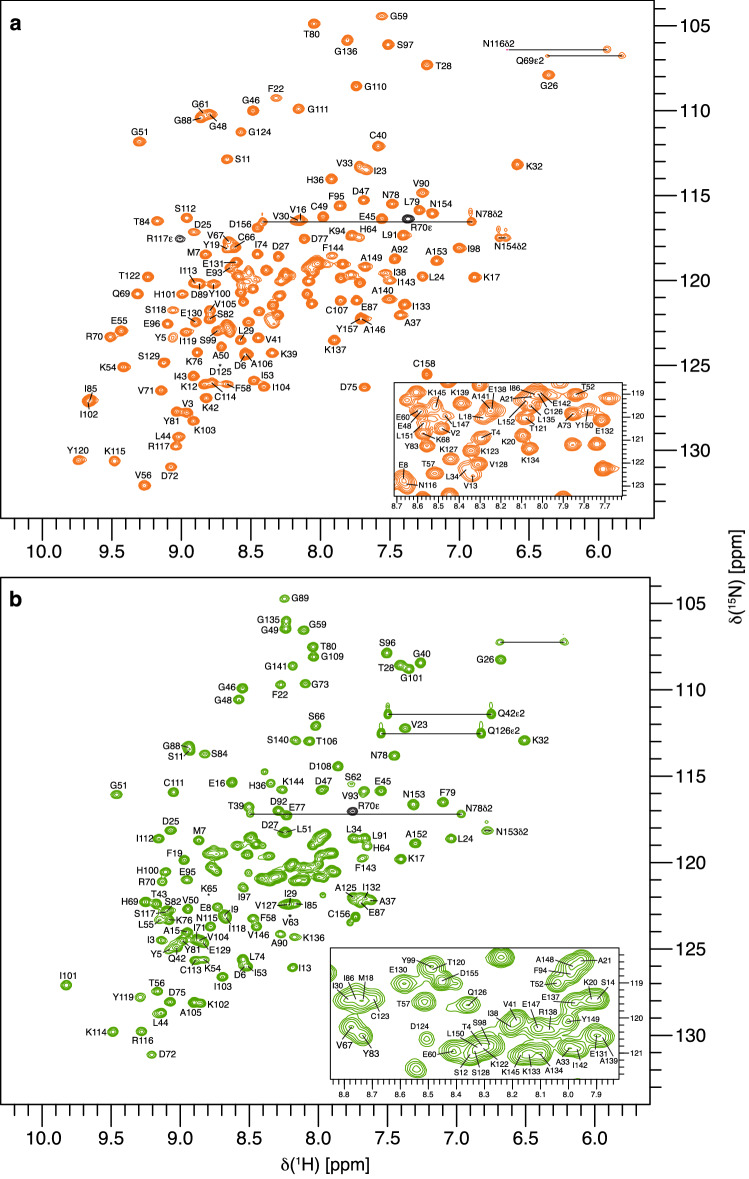
700 MHz ^1^H-^15^N-HSQC of **a** Act c 8.0101, and a 500 MHz ^1^H-^15^N-HSQC **b** Act d 8.0101 (each 0.5 mM) in 20 mM sodium phosphate (pH 6.9), supplemented with 10% D_2_O at 25 °C. Assigned residues are indicated by single letter codes and horizontal lines indicate asparagine and glutamine NH_2_ side-chain resonances. Asterisks indicate the positions of residues below the intensity cut-off. Resonance assignments are available online at the BMRB repository (accession number for Act d 8.0101 is 50811 and 50812 for Act c 8.0101)

We were able to assign the backbone amide resonances of 145 (out of 151) non-proline residues for Act c 8.0101 (Fig. 1a) and 146 (out of 150) non-proline residues for Act d 8.0101 (Fig. 1b). The ^1^H-^15^N-HSQC spectra of the two allergens are indicative of well folded proteins in solution (Table 1).

**Table 1:** Completeness of backbone and side-chain resonance assignments for the two PR-10 allergens from kiwifruit

	Act c 8.0101 (%)	Act d 8.0101 (%)
C’	93.0	99.4
C^α^	97.5	99.4
C^β^	97.3	100.0
C^γ^	73.0	72.5
C^δ^	69.5	79.1
C^ε^	72.2	87.5
H^N^	96.7	98.0
H^α^	92.4	94.5
H^β^	94.3	95.7
H^γ^	78.7	81.7
H^δ^	72.2	90.1
H^ε^	79.7	86.8
N	96.0	97.3
N^δ^	42.9	28.6
N^ε^	42.9	33.3

Assignment for backbone amides H^N^/N corresponds to non-proline residues

**Fig. 2:**
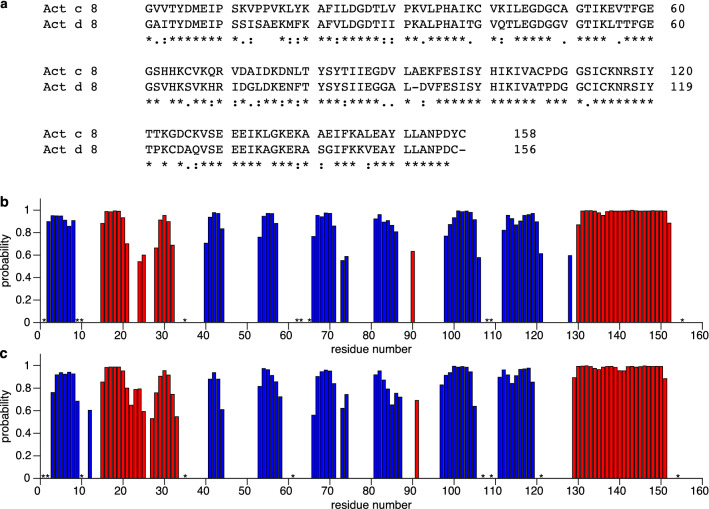
**a** Sequence alignment of Act c 8.0101 and Act d 8.0101. Identical residues in the two proteins are marked by asterisks, while dots indicate different residues with weakly similar properties and colons indicate different residues with strongly similar properties (generated by Clustal Omega (Madeira 2019)). Secondary structure of **b** Act c 8 and **c** Act d 8 as predicted by TALOS+, based on H^N^, N, C’, C^α^, and C^β^ chemical shifts. Secondary structure probabilities (red: α-helices; blue: β-strands) are plotted versus residue numbers. Asterisks indicate residues for which backbone amide H^N^/N resonance assignments are not available

The accession numbers at the Biological Magnetic Resonance Data Bank (http://www.bmrb.wisc.edu) are 50811 for Act d 8.0101 and 50812 for Act c 8.0101. We used the TALOS + software (Shen et al. 2009) to predict the secondary structure elements of the two allergens based on their H^N^, N, C’, C^α^ and C^β^ backbone chemical shifts. Although both allergens only share a sequence identity of 70%, illustrated by the sequence alignment (Fig. 2a), the NMR chemical shifts for both Act c 8 (Fig. 2b) and Act d 8 (Fig. 2c) agree with the canonical PR-10 secondary structure, containing seven β-strands (β1–β7), interrupted by two short α-helices (α1 and α2), and a long α-helix at the C-terminus.

The NMR resonance assignments of Act c 8 and Act d 8 obtained in this work will enable us to analyze structural and dynamic properties of these proteins in a comparative manner, and to investigate their immunological cross-reactivity in detail.
